# Molecular Profiling of Innate Immune Response Mechanisms in Ventilator-associated Pneumonia

**DOI:** 10.1074/mcp.RA120.002207

**Published:** 2020-11-25

**Authors:** Khyatiben V. Pathak, Marissa I. McGilvrey, Charles K. Hu, Krystine Garcia-Mansfield, Karen Lewandoski, Zahra Eftekhari, Yate-Ching Yuan, Frederic Zenhausern, Emmanuel Menashi, Patrick Pirrotte

**Affiliations:** 1 Collaborative Center for Translatinal Mass Spectrometry, Translational Genomics Research Institute, Phoenix, Arizona, USA; 2 Translational Genomics Research Institute, Phoenix, Arizona, USA; 3 HonorHealth Clinical Research Institute, Scottsdale, Arizona, USA; 4 Applied AI and Data Science, City of Hope Medical Center, Duarte, California, USA; 5 Center for Informatics, City of Hope Medical Center, Duarte, California, USA; 6 Center for Applied NanoBioscience and Medicine, University of Arizona, Phoenix, Arizona, USA

**Keywords:** clinical proteomics, metabolomics, immunology, host-pathogen interaction, infectious disease, endotracheal aspirate, metabolome, neutrophil degranulation, proteome, ventilator-associated pneumonia

## Abstract

Ventilator-associated pneumonia (VAP) is a common hospital-acquired infection, leading to high morbidity and mortality. Currently, bronchoalveolar lavage (BAL) is used in hospitals for VAP diagnosis and guiding treatment options. Although BAL collection procedures are invasive, alternatives such as endotracheal aspirates (ETA) may be of diagnostic value, however, their use has not been thoroughly explored. Longitudinal ETA and BAL were collected from 16 intubated patients up to 15 days, of which 11 developed VAP. We conducted a comprehensive LC–MS/MS based proteome and metabolome characterization of longitudinal ETA and BAL to detect host and pathogen responses to VAP infection. We discovered a diverse ETA proteome of the upper airways reflective of a rich and dynamic host-microbe interface. Prior to VAP diagnosis by microbial cultures from BAL, patient ETA presented characteristic signatures of reactive oxygen species and neutrophil degranulation, indicative of neutrophil mediated pathogen processing as a key host response to the VAP infection. Along with an increase in amino acids, this is suggestive of extracellular membrane degradation resulting from proteolytic activity of neutrophil proteases. The metaproteome approach successfully allowed simultaneous detection of pathogen peptides in patients' ETA, which may have potential use in diagnosis. Our findings suggest that ETA may facilitate early mechanistic insights into host-pathogen interactions associated with VAP infection and therefore provide its diagnosis and treatment.

## Teaser

Unlike bronchoalveolar lavages (BAL), the molecular composition of more readily accessible endotracheal aspirates (ETA) has been poorly studied in the context of ventilator-associated pneumonia. In this work, longitudinal ETA and BAL collections from patients under mechanical ventilation were subjected to proteomics and metabolomics analysis. Our integrative omics approach identified signatures of neutrophil mediated induction of host-innate immunity in response to pathogens a day prior to clinical diagnosis.

## Graphical Abstract

**Figure:**
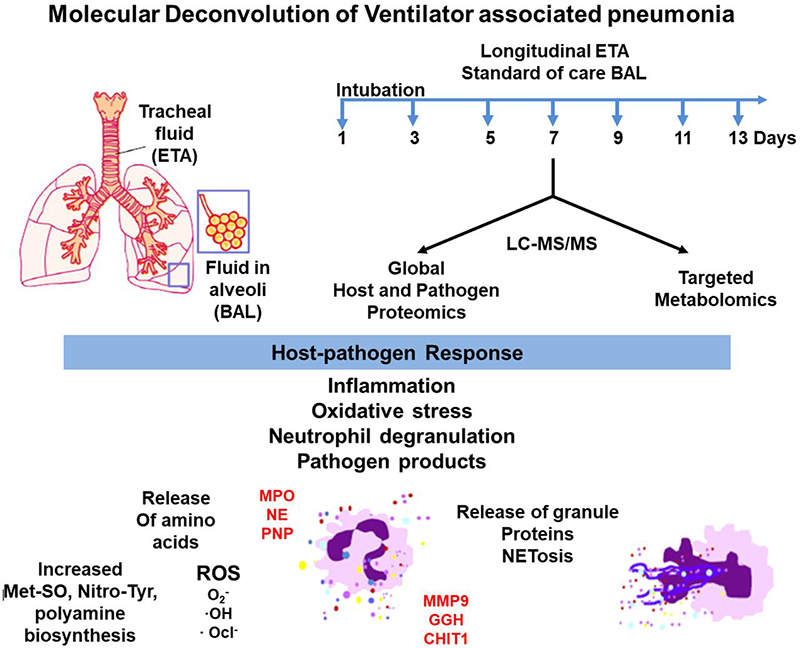

**Highlights**

• ETA present a diverse proteome and metabolome and can be employed for longitudinal studies of nosocomial infections affecting the lungs.
• The proteome and metabolome of ETA and BAL share comparable features that may be leveraged for diagnostics.
• ETA carries early signatures of host innate immunity against ventilator-associated pneumonia.

Ventilator-associated pneumonia (VAP) is the second most common hospital-acquired infection (HAI) in intensive care units (ICU), and is associated to 60% of all HAI-related deaths in the United States (1). It occurs at least 48 h after mechanical ventilation and accounts for more than 300,000 cases annually in the United States. (1, 2). Mechanical ventilation can injure tracheal epithelia and promote environmental microbial colonization and migration from the upper to lower airways (3). All ICU patients with prolonged hospitalization are at high risk of developing VAP, increasing the cost of hospital stay by $40,000 to $50,000 per patient (3, 4, 5). VAP diagnosis is largely based on clinical criteria such as fever, infiltrate on chest radiograph, leukocyte counts, and positive cultures from bronchoalveolar lavages (BAL) (2, 6). With trauma patients, these symptoms are often nonspecific and lead to overestimation of true VAP episodes resulting in the prescription of inadequate broad-spectrum antibiotics (6, 7). A study involving 300 U.S. hospitals showed a 52% increase in antibiotic consumption rate in intensive care unit (ICU) compared with noncritical care (8). In addition, VAP accounts for over half of the antibiotics used in the ICU, which may lead to multi-drug resistance (9). Culture-based testing can reduce antibiotic misuse, but it is time-consuming and can delay diagnosis and treatment. Therefore, careful investigation of VAP pathogenesis is required to better understand host response to microbial dysbiosis and provide insights into the molecular mechanisms underlying the progression of infection.

Previous studies have proposed several candidate biomarkers (interleukin-1β, interleukin-8, soluble triggering receptor expressed on myeloid cells type 1, C-reactive protein (CRP), procalcitonin, and the mid-region fragment of pro-adrenomedullin) to assist VAP diagnosis in serum, plasma or BAL (10, 11, 12, 13). Of these, interleukin-1β and interleukin-8 have been successfully validated in BAL for VAP diagnosis (13). Also, most of these proteins are inflammation markers and have shown variable sensitivity and specificity toward VAP detection (12, 14). This raises potential issues of misdiagnosis and emphasizes the need for further research on specific VAP biomarkers.

BAL has been a widely accepted matrix to study pulmonary infections (15, 16). Many studies have demonstrated the use of BAL for microbial culture, 16S rDNA analysis and determining host-response against VAP infection (16, 17, 18, 19). Endotracheal aspirate (ETA) is regarded as a source for noninvasive respiratory sampling and recently has been recommended for semi quantitative cultures in VAP diagnosis (20). However, the molecular composition of ETA has not been explored as extensively as BAL to understand host responses to infection. We hypothesize that reduced invasiveness involved in ETA sampling is permissive to more frequent longitudinal molecular snapshots of host immune response and changes to microbial flora during early infection. We anticipate that this enhanced granularity provides valuable mechanistic insights into VAP pathogenesis. We used a multi-disciplinary approach integrating proteomics and quantitative metabolomics on longitudinal ETA and matched BAL collected from intubated patients for this study.

## EXPERIMENTAL PROCEDURES

##### Chemicals

Chemicals and solvents were procured from Sigma-Aldrich (St. Louis, MO) or Fisher Scientific (San Jose, CA) unless otherwise stated. The chemicals used in this study were AR grade, and the formic acid (FA) and solvents were LC–MS grade.

##### Specimen Collections

**Fig. 1:**
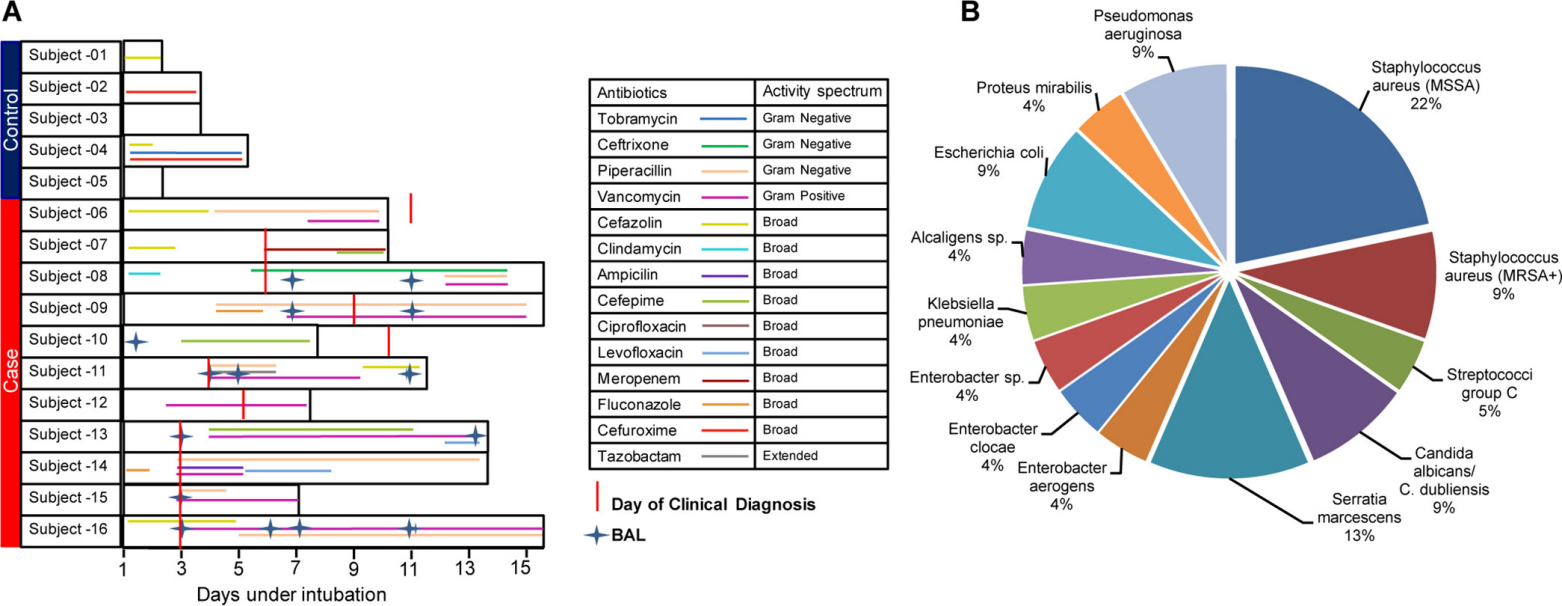
*A*, Patient cohort, sample collections and antibiotic treatment. Sixteen intubated patients enrolled in the study and were categorized in the control and case groups based on clinically diagnosed ventilator-associated pneumonia (VAP). The duration of intubation is denoted by the length of black outlined box for each patient, whereas the duration of antibiotic treatment is denoted by the length of colored line and the type of antibiotic treatment is provided in the legend. The endotracheal aspirate (ETA) samples were collected every other day throughout the intubation period. The bronchoalveolar lavage (BAL) samples were collected as indicated. *B*, Percentage of VAP pathogens detected in BAL culture in clinically diagnosed patients. MSSA = methicillin-sensitive *Staphylococcus aureus*, MRSA+ = methicillin-resistant *S. aureus*.

Patients under mechanical ventilation at the ICU trauma center at HonorHealth Osborne Medical Center, Scottsdale, AZ were enrolled for this study. A written informed consent was obtained from either patient or a legal relative. The clinical protocol for sample collection was approved by the hospital's Institutional Review Board and the Western Institutional Review Board, Puyallup, WA. All experimental procedures conformed to the principles set out in the Declaration of Helsinki and the Department of Health and Human Services Belmont Report. Patients with positive clinical symptoms (≥ 48 h of intubation, fever > 38.4 °C), increases in purulent secretions, new or progressive pulmonary infiltrates on chest radiograph), and a positive culture test using BAL for pathogenic microflora (*Escherichia coli*, *Pseudomonas aeruginosa*, *Staphylococcus aureus*, *Serratia marcescens*, *Streptococci* group C, *Enterobacter cloacae*, *Enterobacter aerogenes*, *Proteus mirabilis*, or *Candida albicans*) were diagnosed with VAP. Clinical diagnosis of VAP was supported by BAL microbial culture tests. Two cut-offs were employed to determine test positivity. A cut-off of 10,000 to 100,000 CFU (colony forming units) was interpreted as presence of a predominant single pathogen, whereas >100,000 CFU was interpreted as presence of more than one potential pathogen. The chest x-ray was performed on all the patients enrolled in the study. The patients with no signs of clinical symptoms were referred as control. ETA was collected every other day, starting at the first day of intubation, until extubation. Upon positive clinical symptoms, BAL was collected as part of standard-of-care procedures (Fig. 1) and used for microbial cultures to aid in clinical diagnosis. BAL collection was done by introducing 50 cc of sterile normal saline to the bronchial lumen to remove mucus plugs, secretions, and debris. The detail description of study cohort and longitudinal collections are summarized in Table I. Both the ETA and BAL biospecimens were collected in sterile tubes and immediately frozen and stored at −80 °C at the collection site. The samples were thawed, filter sterilized with 0.45 μm Ultrafree-CL HV centrifugal filters (Millipore, Billerica, MA), and stored at −80 °C until further processing. In this study, *Baseline* was defined as the first day of intubation for both control and VAP patients, and *VAP positive* as the day of VAP diagnosis. ETA collected 2 days before or after VAP diagnosis was defined as *pre-VAP* and *post-VAP*, respectively. Other time points in control groups were defined as *Control*. This classification was employed for both proteomics and metabolomics data analysis.

**Table I:** Cohort characteristics

Patient-ID	Group	Age	Sex	Days Intubated	ETA^a^	BAL^b^	Culture Test^c^	Clinical VAP^d^
1	Control	25	M	1	1	—	—	N
2	Control	73	M	3	2	—	—	N
3	Control	44	F	3	3	—	—	N
4	Control	33	F	5	1	—	—	N
5	Control	91	F	1	1	—	—	N
6	Case	50	M	9	5	—	+ (11)	Delayed
7	Case	26	M	9	5	—	+ (6)	Y
8	Case	65	F	13	7	2	+ (6)	Y
9	Case	32	F	13	7	2	+ (11)	Y
10	Case	80	F	7	4	1	+ (9)	Delayed
11	Case	70	M	11	6	1	+ (4)	Y
12	Case	71	M	7	4	—	+ (6)	Y
13	Case	77	M	13	7	2	+ (3)	Y
14	Case	75	M	13	7	—	+ (3)	Y
15	Case	79	M	13	7	1	+ (3)	Y
16	Case	56	M	15	7	4	+ (3)	Y


ETA = endotracheal aspirate, BAL = bronchoalveolar lavage,

a ,^b^number of ETA or BAL collections performed for each patient, respectively;

c values in parenthesis indicate the day of intubation corresponding to a positive culture test;

d patients diagnosed with pneumonia after intubation period are categorized as “Delayed”.

##### Proteomics Analysis

ETA and BAL were concentrated using Amicon ultra-3kDa centrifugal filters (Sigma-Aldrich) using the vendor's protocol. The protein concentrations were measured using the Pierce BCA assay kit (ThermoFisher Scientific, San Jose, CA). Various methodologies for preparing BAL and ETA samples for shotgun proteomics analysis were evaluated and are summarized in supplemental Methods S1. As immunodepletion of BAL and ETA yielded superior protein identification (∼708 unique proteins, at least −2.5 fold) compared with other methods (in-gel digestion, in-solution digestion and nanoparticle capture/elution) from both ETA and BAL (supplemental Fig. S1), this approach was selected for sample preparation of both biofluids. The total amount of protein recovered from ETA and BAL were ranging between 160 µg and 406 µg. Equal protein amounts (160 µg) of each BAL or ETA sample were immunodepleted using Pierce Top 12 Abundant Protein Depletion spin columns (ThermoFisher). The flow-through protein solution was subjected to a modified filter-aided sample preparation (21).

Briefly, the samples were buffer exchanged to 50 mm ammonium bicarbonate buffer, pH 7.8, and the proteins were denatured using 8 M urea (1 h), reduced using 1 mm DTT (1 h, 37 °C), and alkylated with 40 mm iodoacetamide (1 h, 37 °C). The protein recovery after immunodepletion was between 5 µg and 35 µg. This variable protein recovery can be explained by the dynamic concentration range of ETA and BAL proteomes, different amounts of high abundance proteins such as Igs, macroglobulin, and transferrin at different stages of infection, and the matrix dilution effect as a result of the saline wash during BAL collections. The proteins were digested with Trypsin Gold overnight at 37 °C (Promega, Madison, WI). The resulting peptides were desalted using C18 SPE cartridges (Waters, Milford, MA) and eluted with 70% acetonitrile (ACN) with 0.1% trifluoroacetic acid (*v/v*) (Waters). The eluted peptides were vacuum-dried and frozen at −20 °C until LC–MS/MS analysis. On the day of analysis, peptide samples were reconstituted in 0.1% formic acid and quantitated using BCA assay. The recovery of peptides after trypsin digestion ranged from 2 µg to 20 µg. The variance in the recovery of immunodepleted proteins from both ETA and BAL limited our ability to perform technical replicates. Sample preparation and data acquisition were randomized separately to minimize bias. LC–MS/MS analysis was performed using a nanoAcquity ultra performance liquid chromatography system (Waters) coupled to an Orbitrap Fusion Lumos Tribrid mass spectrometer (Thermo Fisher). One µg of peptides from each sample was separated on a BEH C18, 1.7 μm, 0.1 × 100 mm column (Waters) using a 83.5 min gradient from 3 to 90% solvent B (ACN, 0.1% FA) and 97 to 10% solvent A (Water, 0.1% FA) at a flow rate of 0.5 µL/min. The following gradient conditions were employed: 3 to 7% B for 1 min, 7 to 25% B for 1 to 72 min, 25 to 45% B for 10 min, 45 to 90% B for 0.5 min, 90% B for 0.5 min and column equilibration at 3% B for 10 min. MS spectra were acquired over a scan range of *m/*z 380 to 2000 using the orbitrap at 120,000 resolution followed by quadrupole isolation (width 1.6 TH) of precursor ions for data-dependent higher-energy collisional dissociation MS/MS with top speed, target automatic gain control values of 50,000, a 60 milliseconds maximum injection time and dynamic exclusion of 60 s. Precursors with charge states of +2 to +7 were fragmented at a normalized collision energy of 35% in the ion trap. *E. coli* tryptic digest (Waters) (250 ng on column) were injected before and after each batch and brackets of 5 samples to assess inter-run variability. Raw data were searched with the Mascot search engine (MatrixScience, Boston, MA) against a recent human database (2015) containing 42,150 protein entries on Proteome Discover v2.1.1.21 (Thermo Scientific) using the following parameters: trypsin rules, maximum 2 missed cleavages, fixed cysteine carbamidomethylation (+57.021 Da) and variable methionine oxidation (+15.995 Da). The precursor and product ion mass tolerances were set to 10 ppm and 0.5 Da, respectively. The target false discovery rate was set to 1%. Peptide quantitation was performed using label-free quantitation based on precursor ion area under the curve (AUC). Percolator in Proteome Discoverer was used to calculate the false-discovery rate with a strict threshold of 0.01 and a relaxed threshold of 0.05 for peptides and PSMs.

For microbial proteomics, we curated a list of common VAP pathogens comprised of Gram-positive, Gram-negative bacteria and yeast (supplemental Table S1) from literature. Proteome FASTA files of the listed pathogens (Gram-positive bacterial and yeast pathogens: 397,142 protein entries, Gram-negative bacterial pathogens: 4,760,935 protein entries) were used as microbiome database and proteomics raw data generated in this study were searched using Mascot with above mentioned parameters. PSM scan numbers for pathogen specific peptides were compared with those matching human peptides to check for overlaps that might be because of PTMs. Any peptides common between human and VAP pathogens were assigned to human peptides only. Peptides specific to bacteria and yeast were maintained for downstream analysis. Peptide sequences from the metaproteomics data were further verified using UniPept v4.0 (22) for their specificity toward microbial pathogens and to perform taxonomic characterization.

For validation of MS observations, MPO (ab119605) and ELANE (ab11955) levels were measured by ELISA (Abcam, Cambridge, MA) in both VAP and control ETA samples according to instructions from the manufacturer.

##### Targeted Metabolomics Analysis

The AbsoluteIDQ® p180 kit (Biocrates Life Sciences AG, Innsbruck Austria) was used to quantify 185 metabolites. Extractions and data analysis were performed using the vendor's instructions. Briefly, metabolites from 10 µL of BAL or ETA were subjected to derivatization using phenylisothiocyanate. The derivatized metabolites were extracted using 5 mm ammonium acetate in methanol followed by solvent extraction. A sample pool of ETA and BAL collections were prepared and used as sample quality control (QC). Triplicate QCs were distributed equally through the run sequence. Data acquisition was conducted on an Acquity UPLC coupled with a Xevo TQ-S mass spectrometer (Waters). For all ETA and BAL, simultaneous quantitation was performed for the following: 21 amino acids, 21 biogenic amines, 40 acylcarnitines, 89 glycerophospholipids. The data were analyzed using MetIDQ™. All the concentrations were measured in micromolar unit. Following nomenclatures, lysophosphatidyl glycerophospholipids (LysoPC x:y), glycerophospholipids (PC aa x:y and PC ae x:y) and Sphingolipids (SM x:y, SM[OH] x:y) were used throughout this manuscript. The x represents as a number of carbons in side chain and y denotes number of unsaturated fatty chains. For fatty acids, “aa” and “ae” represented fatty acids with glycerol moiety and fatty acid with fatty alcohol and glycerol, respectively.

##### Experimental Design and Statistical Rationale

The experimental design comprised longitudinal ETA and BAL collections from 16 intubated patients (11 VAP patients, and 5 controls as detailed in Table I). For the VAP group, 3 to 8 longitudinal ETA samples were collected per patient, whereas in the control group, up to 3 ETA samples were collected per patient (total of 74 ETA and 13 BAL samples). Because of previously described sample limitations, technical replicates were not performed. As patients underwent standard of care, randomization was not performed during collection. All samples were randomized for processing and data acquisition. Relative protein abundances were log2 transformed. In VAP patients, only proteins present in 80% of either *Baseline* or *VAP positive* were selected for downstream proteomic analysis. Abundance of these proteins was also compared with *Baseline* from control patients. For quantitative metabolomics, only metabolites with <20% coefficient of variation (CV) in measurement of QC samples were included for downstream analyses. Metabolites with >50% missing values were removed from the analysis and the remaining missing values were subjected to multivariate imputation by chained equations (MICE) (24). Temporal clustering was performed using median protein abundance or median concentration of metabolites. The distribution of proteomics and metabolomics data were tested using the Shapiro-Wilk test. Both data sets were not normally distributed (*p* > 0.05) and therefore subjected to nonparametric analysis using the Wilcoxon rank-sum test. The p-values were adjusted using the Benjamini-Hochberg *post hoc* test. The following sample groups were compared: *VAP positive* to *Baseline*, *pre-VAP* to *Baseline* and *post-VAP* to *Baseline*. Proteins or metabolites with *p* < *0.05 or adj. p* < *0.05* were considered as significantly different. Gene Ontology (GO) annotation was performed using ToppFun (25). Pathway analysis was performed using Reactome and Ingenuity Pathway Analysis (IPA, Qiagen Inc.) (26, 27). The *p-*values and fold-changes for ETA proteins were input into IPA and mapped against the human Ingenuity Knowledgebase with default values to uncover enriched pathways in VAP patients. The activation z-score was calculated by IPA software to determine positive or negative enrichment of pathways, diseases, and biological functions as categories. The score predicts the increase or decrease in form of positive or negative z-score, respectively. The proteomics and metabolomics data were further assessed for similarity between ETA and BAL matrices using Bland-Altman analysis (28).

## RESULTS

##### Study Cohort

Our study cohort was composed of 16 trauma patients intubated up to 15 days. Eleven of these patients exhibited symptoms of pneumonia (VAP patients) and five did not present any signs of pulmonary infection (control patients). The clinical annotation and antibiotic regimens are described in Table I and Fig. 1*A*. Duration of intubation was longer in VAP (≥ 7 days) than control patients (≤ 5 days). A total of 8 patients including 3 control patients and 5 VAP patients were given broad spectrum antibiotics at intubation, whereas no antibiotics were given to the remaining 2 controls and 6 VAP patients. In VAP patients, antibiotic prophylaxis at the time of intubation did not show any better protection compared with no antibiotics; also, there was no clear-cut effect of antibiotic prophylaxis on the length of mechanical ventilation. Further antibiotic treatment was aligned as per BAL culture and clinical diagnosis. Based on BAL culture, *Staphylococcus aureus* was the most common VAP pathogen observed: 7 patients harbored methicillin-sensitive *S. aureus* (MSSA) and 2 patients harbored methicillin-resistant *S. aureus* (MRSA) (Fig. 1*B*). As all patients in our study cohort were administered a standard-of-care regimen of antibiotics, their impact on the patient proteome and metabolome was not evaluated.

##### ETA Proteome Reveals Neutrophil Mediated Response in VAP

**Fig. 2:**
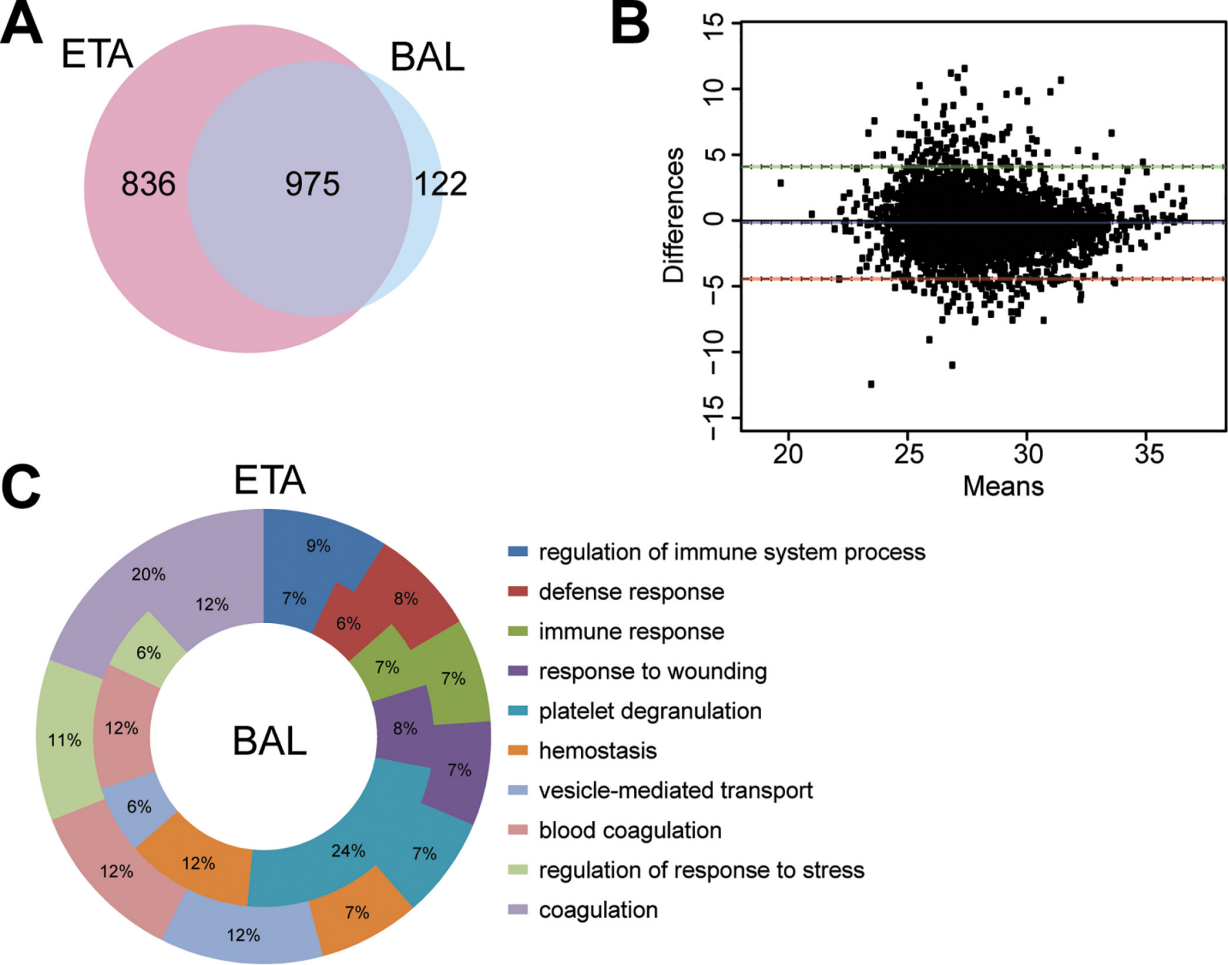
**Comparison of ETA and BAL proteomes in VAP patients.***A*, The Venn diagram shows the numbers of proteins shared between ETA and BAL proteins. *B*, Bland-Altman analysis of bias and 95% limit of agreement for ETA *versus* BAL proteins. *C*, Gene ontology (GO) analysis (biological process) of ETA and BAL proteins. donut plot shows the top 10 enriched GO terms of biological process for BAL (inner core) and ETA (outer core) proteins.

We identified a total of 3067 unique proteins in ETA collections across all patients and time points. We compared patient-matched ETA and BAL collected on the same day and identified 1811 and 1097 unique proteins in ETA and BAL collected on the same day. Of these, 975 proteins represented 88.9% of BAL proteome of this cohort and mapped to 187 significant reactome pathways. The top 10 mapped pathways were *neutrophil degranulation, innate immune system, immune system, complement cascade, regulation of complement cascade, platelet activation, signaling and aggregation, platelet degranulation, regulation of insulin-like growth factor transport, post-translational protein phosphorylation and hemostasis* (supplemental Table S2). These suggest enrichment of proteins associated to host immunity in both ETA and BAL. Further, Bland-Altman comparison of these shared proteins showed that there was no significant bias between ETA and BAL as most of the data sits between the 95% confidence interval upper limit of 4.1 and the lower limit of −4.4. (Fig. 2*B*). The GO terms for the biological processes related to innate and humoral immunity were similarly enriched across both fluids (Fig. 2*C*). This suggests similarities in proteome composition between BAL and ETA.

Because of the relative ease-of-access and increased sampling availability, we focused our study on ETA. Because intubation was variable across VAP patients and controls, we compared the first day of intubation (*Baseline*) against subsequent time points in VAP patients and compared *Baseline* ETA proteome in VAP and control patients.

**Fig. 3:**
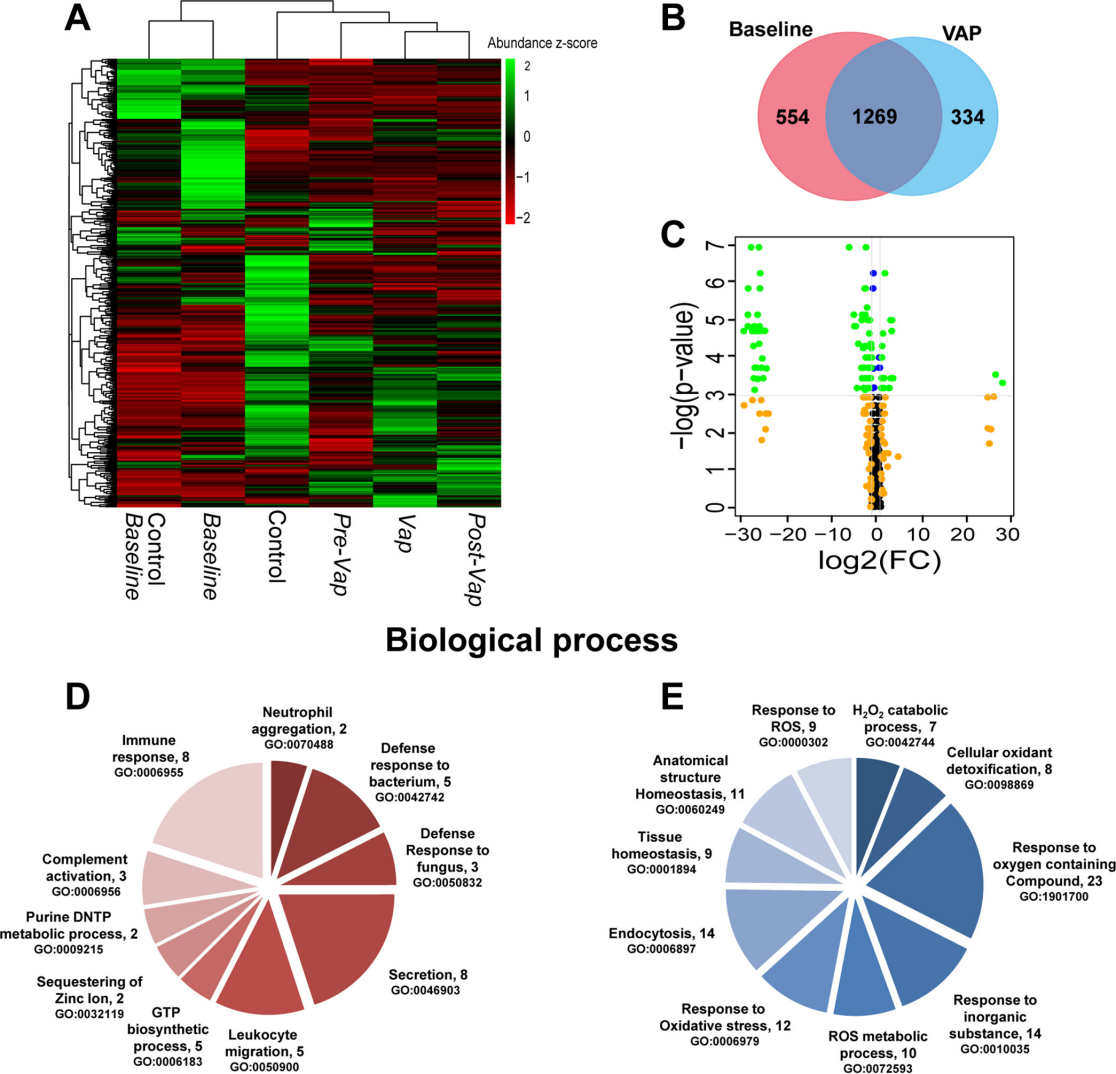
**ETA proteome analysis.***A*, Temporal clustering of clinical time points using median protein abundance. *B*, Comparison of the *Baseline* and *VAP positive* ETA proteome in VAP patients. *C*, Significantly upregulated proteins in VAP patients*;* Volcano plot representing the ETA proteome with, (1) in blue, significant proteins (*p* < 0.05, Wilcoxon rank-sum test), (2) in orange, proteins for which the log2(FC) of the abundance (*VAP positive* compared with *Baseline*) > 1 or < −1, and (3) in green, significant proteins (*p* < 0.05) with log2(FC) > 1 or < −1 FC = Fold change. *D*, *E*, Gene Ontology (GO)-based functional enrichment analysis of significantly differentially abundant proteins in *VAP positive* compared with *Baseline* (biological process). Upregulated (*left*) and downregulated (*right*) GO categories (parenthesis). *Baseline* = day 1 of intubation for Control and VAP, *VAP positive* = day of VAP diagnosis, *pre-VAP* = a day before VAP, *post-VAP* = a day after VAP, *Control* = other intubation time points in control patients (day3 and day5).

The patients enrolled in our study were intubated for variable length of time and developed infection at different days. We categorized longitudinal collections to major clinical events of interest, such as *Baseline*, *VAP positive*, *pre-VAP* and *post-VAP*. Binning median protein abundance for each event, unsupervised temporal clustering showed concomitant clustering of *Baselines* of both control (Control *Baseline*) and VAP patients (VAP *Baseline*), suggesting that at the time of intubation, ETA proteomes of both control and VAP patients remained unchanged (Fig. 3*A*). The ETA proteome in VAP patients was following the pattern of progression of VAP infection from *pre-VAP, VAP positive* and *post-VAP* (Fig. 3*A*).

In VAP patients, a total of 1823 and 1603 proteins were identified in the *Baseline* and *VAP positive* ETA, respectively, with 1269 proteins shared between time points (Fig. 3*B*). Out of those, 10 to 19% of unique proteins were identified across all ETA, reflecting proteome variability in longitudinal samples.

The 1269 shared ETA proteins were used to compare the *Baseline* and *VAP positive* in VAP patients. Changes were further compared with *Pre-VAP* and *Post-VAP* groups. Ingenuity Pathway Analysis (IPA) on all proteins between *VAP positive* and *Baseline* revealed 133 enriched pathways. *Complement system*, *Acute phase response signaling*, *Actin cytoskeleton signaling*, *Gluconeogenesis I*, *Integrin signaling*, *Glycolysis I*, *Regulation of actin-based motility by Rho*, and *Pentose phosphate pathway* are positively enriched, whereas *LXR/RXR activation* and *Remodeling of epithelial adherens junctions* are negatively enriched (supplemental Table S3). GO terms above were further verified by the biological functions and diseases from IPA, which reported *degranulation of neutrophils, granulocytes,* and *phagocytes*, *leukocyte migration*, *inflammation*, *apoptosis*, and *necrosis* as most enriched (supplemental Table S4). These pathways and processes were also observed to be enriched in *Pre-VAP* samples compared with *Baseline* and suggesting early activation of leukocyte mediated immunity in response to VAP pathogens.

Further statistical comparisons identified 96 differentially abundant proteins (*p* < 0.05, median fold change >2) between *Baseline* and *VAP positive* (Fig. 3*C*) (Table II). Twenty upregulated proteins contributed to the following GO terms (biological processes, *p* < 0.05): *neutrophil aggregation*, *defense response to bacterium and fungus*, *leukocyte migration*, and *complement activation* (Fig. 3*D*); 76 downregulated proteins were linked to *reactive oxygen species metabolic process*, *oxidative stress*, *cellular oxidant detoxification*, and *tissue homeostasis* (Fig. 3*E*). This suggests neutrophil mediated innate immune response and wound healing processes in *VAP positive*.

**Table II:** Differential expression of ETA proteins between Baseline and the day of clinical diagnosis (VAP positive)

UniProt Accession	Protein Name (Gene ID)	log2(FC)^a^	*p* Value
P02741	C-reactive protein (CRP)	28.11	0.0366
P15531-2	Isoform 2 of Nucleoside diphosphate kinase A (NME1)	26.56	0.0293
P27918	Properdin (CFP)	3.68	0.0323
P06702	Protein S100-A9 (S100A9)	3.45	0.0068
O00602	Ficolin-1 (FCN1)	3.39	0.0092
O14950	Myosin regulatory light chain 12B (MYL12B)	3.21	0.0323
P05109	Protein S100-A8 (S100A8)	3.14	0.0068
Q6UX06	Olfactomedin-4 (OLFM4)	2.87	0.0420
P35579	Myosin-9 (MYH9)	2.71	0.0420
Q9HD89	Resistin (RETN)	1.87	0.0020
P14780	Matrix metalloproteinase-9 (MMP9)	1.72	0.0322
Q13451	Peptidyl-prolyl cis-trans isomerase FKBP5 (FKBP5)	1.71	0.0323
Q9HB71	Calcyclin-binding protein (CACYBP)	1.62	0.0420
Q96C19	EF-hand domain-containing protein D2 (EFHD2)	1.53	0.0420
Q13231	Chitotriosidase-1 (CHIT1)	1.43	0.0098
Q92820	Gamma-glutamyl hydrolase (GGH)	1.25	0.0137
P05164-3	Isoform H7 of Myeloperoxidase (MPO)	1.21	0.0186
P00491	Purine nucleoside phosphorylase (PNP)	1.16	0.0420
P54819	Adenylate kinase 2, mitochondrial (AK2)	1.14	0.0322
P18206	Vinculin (VCL)	1.08	0.0420
P07339	Cathepsin D (CTSD)	−1.03	0.0186
P63104	14-3-3 protein zeta/delta (YWHAZ)	−1.11	0.0144
P13987	CD59 glycoprotein (CD59)	−1.13	0.0244
O00299	Chloride intracellular channel protein 1 (CLIC1)	−1.14	0.0244
P17931	Galectin-3 (LGALS3)	−1.19	0.0244
P00746	Complement factor D (CFD)	−1.23	0.0129
P11413-2	Isoform Long of Glucose-6-phosphate 1-dehydrogenase (G6PD)	−1.29	0.0322
P07355-2	Isoform 2 of Annexin A2 (ANXA2)	−1.49	0.0068
P01834	Ig kappa chain C region (IGKC)	−1.62	0.0098
P09668	Pro-cathepsin H (CTSH)	−1.64	0.0186
P80723	Brain acid soluble protein 1 (BASP1)	−1.67	0.0244
O43490	Prominin-1 (PROM1)	−1.70	0.0420
P00558	Phosphoglycerate kinase 1 (PGK1)	−1.71	0.0323
Q08380	Galectin-3-binding protein (LGALS3BP)	−1.82	0.0186
P00915	Carbonic anhydrase 1 (CA1)	−1.85	0.0244
P12273	Prolactin-inducible protein (PIP)	−1.92	0.0186
P43652	Afamin (AFM)	−1.93	0.0068
Q32MZ4-3	Isoform 3 of Leucine-rich repeat flightless-interacting protein 1 (LRRFIP1)	−1.98	0.0323
P69905	Hemoglobin subunit alpha (HBA1)	−1.99	0.0420
P00918	Carbonic anhydrase 2 (CA2)	−2.03	0.0420
P02766	Transthyretin (TTR)	−2.09	0.0049
P01591	Immunoglobulin J chain (IGJ)	−2.19	0.0137
Q9BW30	Tubulin polymerization-promoting protein family member 3 (TPPP3)	−2.35	0.0010
P02765	Alpha-2-HS-glycoprotein (AHSG)	−2.39	0.0186
Q06830	Peroxiredoxin-1 (PRDX1)	−2.43	0.0029
P32119	Peroxiredoxin-2 (PRDX2)	−2.48	0.0059
Q16270	Insulin-like growth factor-binding protein 7 (IGFBP7)	−2.52	0.0323
Q13228-4	Isoform 4 of Selenium-binding protein 1 (SELENBP1)	−2.54	0.0059
P01620	Ig kappa chain V-III region SIE (IGKV3-20)	−2.55	0.0440
Q6P5S2	UPF0762 protein C6orf58 (C6orf58)	−2.59	0.0059
P15311	Ezrin (EZR)	−2.64	0.0029
O14745	Na(+)/H(+) exchange regulatory cofactor NHE-RF1 (SLC9A3R1)	−2.67	0.0420
P61769	Beta-2-microglobulin (B2M)	−2.67	0.0137
P68871	Hemoglobin subunit beta (HBB)	−2.79	0.0420
Q8N4F0	BPI fold-containing family B member 2 (BPIFB2)	−2.80	0.0244
P19823	Inter-alpha-trypsin inhibitor heavy chain H2 (ITIH2)	−2.92	0.0420
P23528	Cofilin-1 (CFL1)	−2.96	0.0144
P30838	Aldehyde dehydrogenase, dimeric NADP-preferring (ALDH3A1)	−3.18	0.0059
P07711	Cathepsin L1 (CTSL)	−3.20	0.0323
P10909-2	Isoform 2 of Clusterin (CLU)	−3.27	0.0068
Q14103	Heterogeneous nuclear ribonucleoprotein D0 (HNRNPD)	−3.36	0.0244
P13667	Protein disulfide-isomerase A4 (PDIA4)	−4.04	0.0129
P02042	Hemoglobin subunit delta (HBD)	−4.32	0.0420
O75347	Tubulin-specific chaperone A (TBCA)	−4.63	0.0080
P02545	Prelamin-A/C (LMNA)	−4.92	0.0080
P23141-2	Isoform 2 of Liver carboxyllesterase 1 (CES1	−5.07	0.0059
Q13938	Calcyphosin (CAPS)	−6.11	0.0010
P13010	x-ray repair cross-complementing protein 5 (XRCC5)	−24.52	0.0248
P08294	Extracellular superoxide dismutase [Cu-Zn] (SOD3)	−24.87	0.0091
Q5RHP9	Glutamate-rich protein 3 (ERICH3)	−24.97	0.0092
P27105	Erythrocyte band 7 integral membrane protein (STOM)	−25.25	0.0323
P09758	Tumor-associated calcium signal transducer 2 (TACSTD2)	−25.30	0.0091
P67936	Tropomyosin alpha-4 chain (TPM4)	−25.46	0.0191
Q15185	Prostaglandin E synthase 3 (PTGES3)	−25.60	0.0244
P34932	Heat shock 70 kDa protein 4 (HSPA4)	−25.93	0.0020
P50502	Hsc70-interacting protein (ST13)	−25.99	0.0029
Q96NY7	Chloride intracellular channel protein 6 (CLIC6)	−26.08	0.0080
P30043	Flavin reductase (NADPH) (BLVRB)	−26.14	0.0092
P06727	Apolipoprotein A-IV (APOA4)	−26.16	0.0129
P43353	Aldehyde dehydrogenase family 3 member B1 (ALDH3B1)	−26.22	0.0010
P30041	Peroxiredoxin-6 (PRDX6)	−26.31	0.0092
P07988	Pulmonary surfactant-associated protein B (SFTPB)	−26.46	0.0330
Q13740	CD166 antigen (ALCAM)	−26.52	0.0059
P19338	Nucleolin (NCL)	−26.60	0.0244
P05452	Tetranectin (CLEC3B)	−27.06	0.0092
P51884	Lumican (LUM)	−27.13	0.0244
P15328	Folate receptor alpha (FOLR1)	−27.16	0.0440
P13647	Keratin, type II cytoskeletal 5 (KRT5)	−27.25	0.0080
Q04917	14-3-3 protein eta (YWHAH)	−27.34	0.0323
P02753	Retinol-binding protein 4 (RBP4)	−27.42	0.0137
P15090	Fatty acid-binding protein, adipocyte (FABP4)	−27.70	0.0092
P08727	Keratin, type I cytoskeletal 19 (KRT19)	−27.95	0.0010
P31946	14-3-3 protein beta/alpha (YWHAB)	−28.61	0.0029
P00352	Retinal dehydrogenase 1 (ALDH1A1)	−28.68	0.0059
P06396	Gelsolin (GSN)	−28.68	0.0080
P05787-2	Isoform 2 of Keratin, type II cytoskeletal 8 (KRT8)	−29.58	0.0092


a log2(FC) of *VAP positive* to *Baseline*, FC = fold change.

To determine response specificity against infection, we compared *Baseline* ETA between VAP and control patients. Of the 96 differentially abundant proteins identified in the longitudinal analysis, YWHAH was significantly higher (*p* < 0.05, 2.4-fold) in VAP *Baseline* compared with control *Baseline*. Further comparison of VAP *Baseline* with other intubation time points in controls showed significant increase of PGK1 (*p* < 0.05, 2-fold) and NCL (*p* < 0.05, 2.2-fold) in the VAP *Baseline*. However, 26 proteins including YWHAH and NCL were not detected in *VAP positive* but were present in all *Baseline* (Fig. 3*B*, Table III). Functional annotation reveals their role in multiple binding activities (*p* < 0.05) (*hormone binding*, *vitamin binding*, *copper ion binding*, *scaffold protein binding*) (Fig. 3*A*, supplemental Table S5). Absence of these proteins in *VAP positive* may imply pathogen binding and clearance. Both isoform 2 of nucleoside diphosphate kinase A (NME-1) and CRP were detected in VAP ETA only (Table III). To gain further insight, the significant differentially abundant proteins were mapped to Reactome pathways (Table IV). Two of the pathways with low *p-*values (*p* < 6.6E-14), *neutrophil degranulation* (11 proteins) and *innate immune system* (13 proteins) represent 55 to 65% of all upregulated proteins, suggesting increased secretion of host immune proteins in VAP patients. Out of 76 downregulated proteins (Fig. 3*C*), 48 proteins were mapped to top 10 significant (*p* < 0.05) pathways (Table IV). The majority of upregulated proteins mapped to multiple pathways linked to pathogen recognition and innate immunity, whereas most downregulated blood proteins, carbonic anhydrase 1 (CA1), carbonic anhydrase 2 (CA2), hemoglobin subunit beta (HBB), hemoglobin subunit alpha (29), hemoglobin subunit delta (HBD), peroxiredoxin-1 (PRDX1), peroxiredoxin-2 (PRDX2), peroxiredoxin-6 (PRDX6) and erythrocyte band 7 integral membrane protein (STOM) mapped to tissue injury (Table II, IV).

**Table III:** Comparison of ETA proteins between Baselines (from control and VAP patients) and VAP positive

Protein Name	Gene ID	log2(FC)^a^	*p* Value	Median Area under Curve (AUC)		
				Control *Baseline*	VAP Patients	
					*Baseline*	*VAP Positive*
Retinal dehydrogenase 1	ALDH1A1	−28.68	0.006	26.96	28.68	0.00
Retinol-binding protein 4	RBP4	−27.42	0.014	28.44	27.42	0.00
Tetranectin	CLEC3B	−27.06	0.009	25.23	27.06	0.00
Isoform 2 of Keratin, type II cytoskeletal 8	KRT8	−29.58	0.009	27.59	29.58	0.00
Gelsolin	GSN	−28.68	0.008	30.26	28.68	0.00
Apolipoprotein A-IV	APOA4	−26.16	0.013	25.07	26.16	0.00
Pulmonary surfactant-associated protein B	SFTPB	−26.46	0.033	25.99	26.46	0.00
Extracellular superoxide dismutase [Cu-Zn]	SOD3	−24.88	0.009	24.53	24.88	0.00
Keratin, type I cytoskeletal 19	KRT19	−27.95	0.001	27.81	27.95	0.00
Tumor-associated calcium signal transducer 2	TACSTD2	−25.30	0.009	20.13	25.30	0.00
x-ray repair cross-complementing protein 5	XRCC5	−24.52	0.025	21.24	24.52	0.00
Keratin, type II cytoskeletal 5	KRT5	−27.25	0.008	25.77	27.25	0.00
Fatty acid-binding protein, adipocyte	FABP4	−27.70	0.009	28.34	27.70	0.00
Folate receptor alpha	FOLR1	−27.16	0.044	26.58	27.16	0.00
Nucleolin	NCL	−26.60	0.024	24.80	26.60	0.00
Peroxiredoxin-6	PRDX6	−26.31	0.009	25.34	26.31	0.00
Flavin reductase (NADPH)	BLVRB	−26.14	0.009	25.21	26.14	0.00
14-3-3 protein beta/alpha	YWHAB	−28.61	0.003	27.98	28.61	0.00
Heat shock 70 kDa protein 4	HSPA4	−25.93	0.002	25.93	25.93	0.00
Hsc70-interacting protein	ST13	−25.99	0.003	25.81	25.99	0.00
Lumican	LUM	−27.13	0.024	25.53	27.13	0.00
Tropomyosin alpha-4 chain	TPM4	−25.46	0.019	26.43	25.46	0.00
14-3-3 protein eta	YWHAH	−27.34	0.032	26.10	27.34	0.00
CD166 antigen	ALCAM	−26.52	0.006	26.37	26.52	0.00
Glutamate-rich protein 3	ERICH3	−24.97	0.009	22.13	24.97	0.00
Chloride intracellular channel protein 6	CLIC6	−26.08	0.008	25.22	26.08	0.00
Peptidyl-prolyl cis-trans isomerase FKBP5	FKBP5	1.71	0.032	0.00	23.39	25.10
Ig kappa chain V-III region SIE	IGKV3-SIV	−2.56	0.044	0.00	28.12	25.57
Ficolin-1	FCN1	3.39	0.009	0.00	22.56	25.95
Isoform 2 of Nucleoside diphosphate kinase A	NME1	26.56	0.029	0.00	0.00	26.56
C-reactive protein	CRP	28.11	0.037	0.00	0.00	28.11


a Log2(FC) of *VAP* positive *versus Baseline* in VAP patients; *p-value* determined using Wilcoxon rank sum test.

**Table IV:** Reactome pathway analysis of differentially expressed proteins between Baseline and VAP positive

Pathway	Significant Proteins (*p* < 0.05*)*	*p* Value
Up Regulated		
Neutrophil degranulation	FCN1;GGH;RETN;CFP;OLFM4;MPO;MMP9;NME1;CHIT1PNP;S100A9;S100A8;VCL	6.59E-14
Innate immune system	FCN1;CRP;GGH;RETN;CFP;OLFM4;MPO;MMP9;NME1CHIT1;PNP;MYH9;S100A9;S100A8;VCL	6.24E-10
Initial triggering of complement	FCN1;CRP;CFP	9.27E-05
Smooth muscle contraction	VCL;MYL12B	6.68E-05
EPH-Ephrin signaling	MYH9;MMP9;MYL12B	5.55E-05
RHO GTPases activate ROCKs, CIT, PAKS	MYH9;MYL12B	9.89E-04
Interconversion of nucleotide di- and triphosphates	AK2;NME1	6.07E-04
Metal sequestration by antimicrobial proteins	S100A9;S100A8	3.20E-04
Ficolins bind to repetitive carbohydrate structures on the target cell surface	FCN1	2.73E-04
Metabolism of nucleotides	PNP;AK2;NME1	1.60E-03
Downregulated		
Erythrocytes take up oxygen and release carbon dioxide	CA1;CA2; HBA1;HBA2;HBB	4.33E-07
Neutrophil degranulation	CD59;PRDX6;LGALS3;XRCC5;CFD;TTR;HSG;GSN;B2M;ALDH2B1;HBB;STOM;CTSD;CTSH	9.58E-06
Chk1/Chk2(Cds1) mediated inactivation of Cyclin B:Cdk1 complex	YWHAB;YWHAH;YWHAZ	1.02E-04
Detoxification of reactive oxygen species	PRDX1;PRDX2;PRDX6;SOD3	2.06E-04
TP53 regulates metabolic genes	PRDX1;PRDX2;YWHAB;YWHAZ;YWHAH	4.92E-04
Neurodegenerative diseases	PRDX1;PRDX2;LMNA	6.77E-04
Scavenging of heme from plasma	HBA1;HBA2;JCHAIN;KappaChainVII-SIE, KappaChainC	8.32E-04
Amyloid fiber formation	TTR;GSN;B2M;APOA4	1.80E-03
Platelet degranulation	LGALS3BP;CFL1;CFD;AHSG;CLEC3B	2.55E-03

**Fig. 4:**
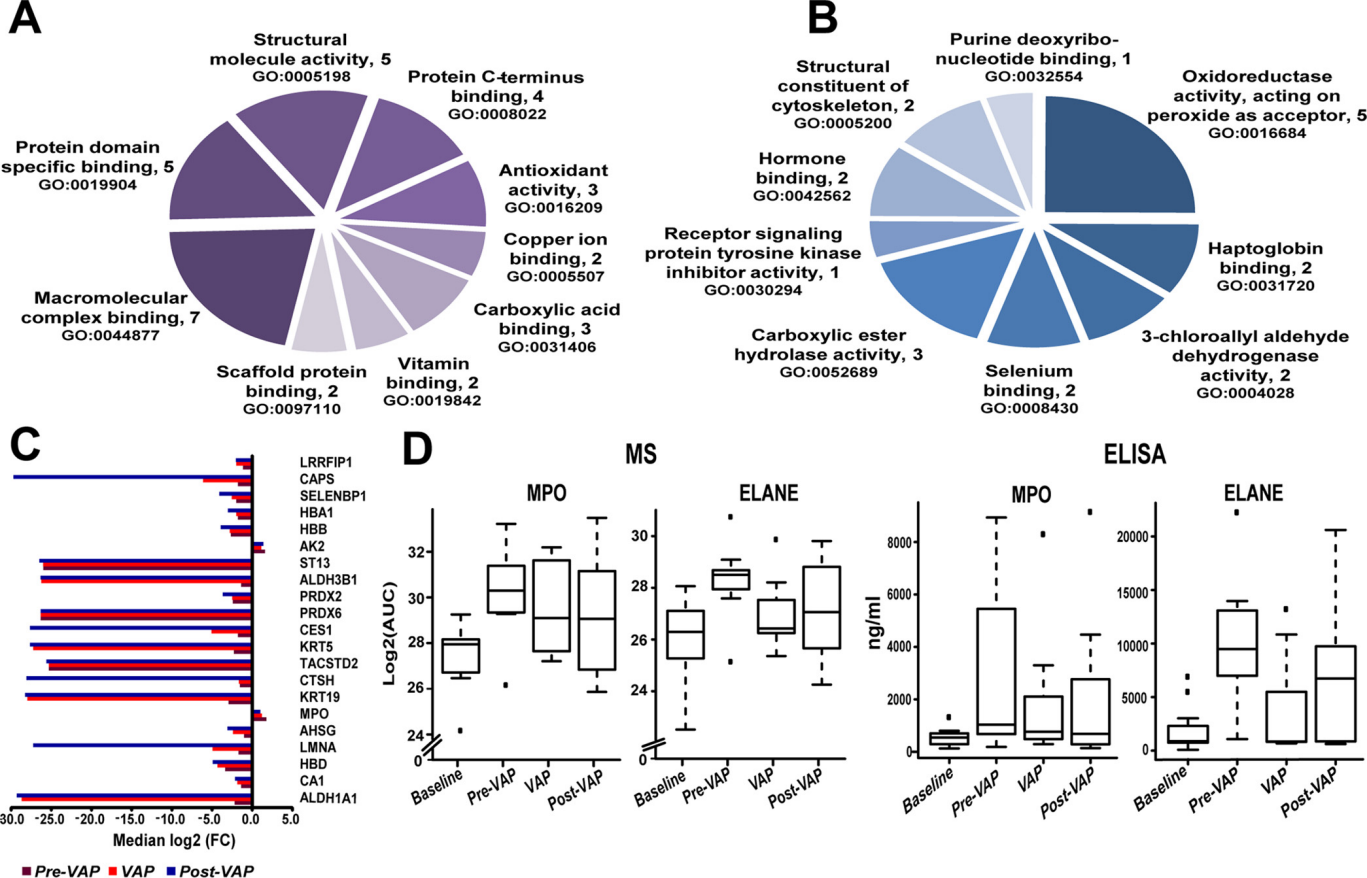
**Analysis of functional enrichment and protein levels of differentially abundant proteins.***A*, GO molecular function analysis of proteins only present in *Baseline* (at day of intubation). *B*, GO molecular function analysis of downregulated proteins in *pre-VAP* and *VAP positive* relative to *Baseline*. *C*, Median log2(FC) of protein abundance for 21 significant proteins in *pre-VAP* (dark *red*), *VAP positive* (*red*), and *post-VAP* (*blue*) compared with *Baseline*. *D*, Measurement of MPO and ELANE levels in VAP patients using MS aided relative abundance, log2(AUC) (*left*) and ELISA (ng/ml of ETA) (*right*).

To identify early VAP response mechanisms, we evaluated ETA collected 2 days prior to clinical diagnosis (*pre-VAP*). We identified 21 significantly differentially abundant proteins (*p* < 0.05) with a fold change >2 compared with *Baseline* (Fig. 3*C*, Table V). Consistent with our prior analysis, each of the 19 downregulated proteins were associated with binding functions suggestive of scavenging and sequestration as an early host response to infection (Fig. 4*A*–*C*). We observed recurrence of binding mechanisms and upregulation of both isoform H7 of myeloperoxidase (MPO) and adenylate kinase 2 (AK2) in *pre-VAP* ETA (Fig. 4*C*). Although MPO has been involved in neutrophil mediated innate immunity (30), AK2 has been reported as a ubiquitous marker of cell lysis (31). This suggests their potential role in early defense against VAP infection, prompting us to explore neutrophil mediated pathogen processing. Longitudinal trends measured by LC–MS/MS of the two key components of neutrophil granules, MPO and ELANE, were confirmed by ELISA (Fig. 4*D*). Both proteins were significantly higher in *pre-VAP* (4.8- to 5-fold compared with *Baseline*, adj. *p* < 0.044) and *VAP positive* (3.4 to 4-fold compared with *Baseline*, adj. *p* < 0.038), highlighting the rapid initiation of neutrophil degranulation and may serve as early detection markers.

**Table V:** Comparison of the ETA proteome among Baseline, VAP positive (day of diagnosis) and *pre-VAP* (2 days prior to diagnosis)

Protein Name	Gene ID	*VAP Positive*		*pre-VAP*	
		log2(FC)^a^	*p* value	log2(FC)^b^	*p* value
Retinal dehydrogenase 1	ALDH1A1	−28.680	0.006	−2.178	0.036
Carbonic anhydrase 1	CA1	−1.854	0.024	−1.375	0.022
Hemoglobin subunit delta	HBD	−4.322	0.042	−3.364	0.022
Prelamin-A/C	LMNA	−4.922	0.008	−1.703	0.036
Alpha-2-HS-glycoprotein	AHSG	−2.389	0.019	−1.022	0.022
Isoform H7 of Myeloperoxidase	MPO	1.206	0.019	1.759	0.022
Keratin, type I cytoskeletal 19	KRT19	−27.954	0.001	−2.935	0.022
Pro-cathepsin H	CTSH	−1.644	0.019	−1.539	0.035
Tumor-associated calcium signal transducer 2	TACSTD2	−25.301	0.009	−25.301	0.036
Keratin, type II cytoskeletal 5	KRT5	−27.253	0.008	−2.261	0.036
Isoform 2 of Liver carboxyllesterase 1	CES1	−5.067	0.006	−1.794	0.036
Peroxiredoxin-6	PRDX6	−26.307	0.009	−26.307	0.036
Peroxiredoxin-2	PRDX2	−2.481	0.006	−2.402	0.022
Aldehyde dehydrogenase family 3 member B1	ALDH3B1	−26.217	0.001	−1.379	0.022
Hsc70-interacting protein	ST13	−25.990	0.003	−25.990	0.022
Adenylate kinase 2, mitochondrial	AK2	1.138	0.032	1.585	0.022
Hemoglobin subunit beta	HBB	−2.790	0.042	−2.657	0.022
Hemoglobin subunit alpha	HBA1	−1.992	0.042	−1.792	0.022
Isoform 4 of Selenium-binding protein 1	SELENBP1	−2.544	0.006	−1.972	0.036
Calcyphosin	CAPS	−6.112	0.001	−1.790	0.022
Isoform 3 of Leucine-rich repeat flightless-interacting protein 1	LRRFIP1	−1.980	0.032	−1.150	0.036


a ,^b^indicates log2(FC) of *VAP positive* to *Baseline* and *pre-VAP* to *Baseline*, respectively.

##### VAP Patients Harbor Metabolic Signatures of Oxidative Stress

**Fig. 5:**
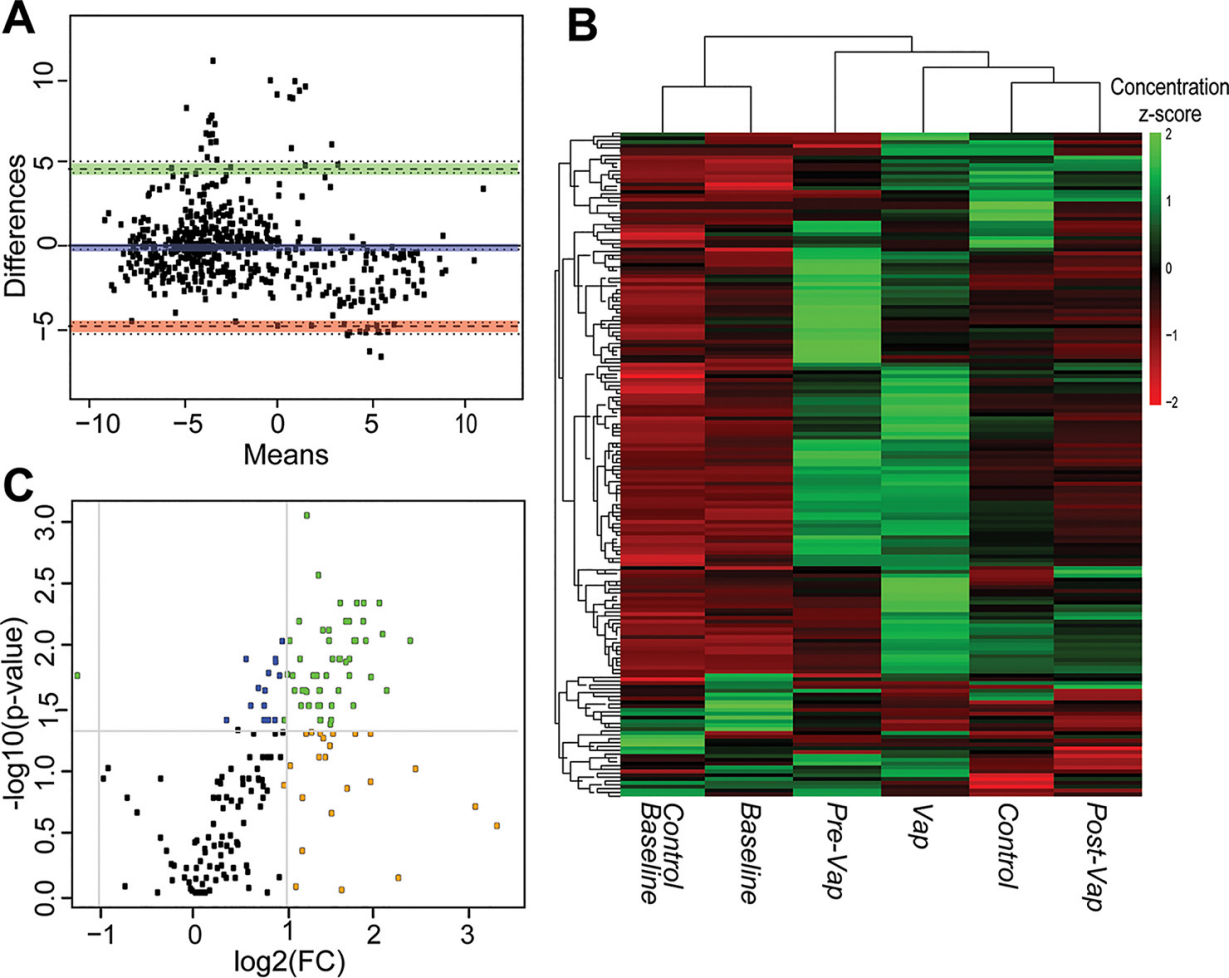
**Differential metabolomic analysis.***A*, Bland-Altman analysis of metabolite concentrations (μm) from ETA and BAL collected on the same day. *B*, Temporal clustering of clinical time points using median concentrations of metabolites in ETA. *C*, The volcano plot indicates differential concentrations of 185 metabolites in ETA by comparing the *VAP positive* to *Baseline*. (1) In blue, significant metabolites, (2) in orange, metabolites for which the log2(FC) of metabolite concentration (*VAP positive* compared with *Baseline*) > 1 or < −1; (3) in green, significant metabolites with log2(ratio) > 1 or < −1.

**Fig. 7:**
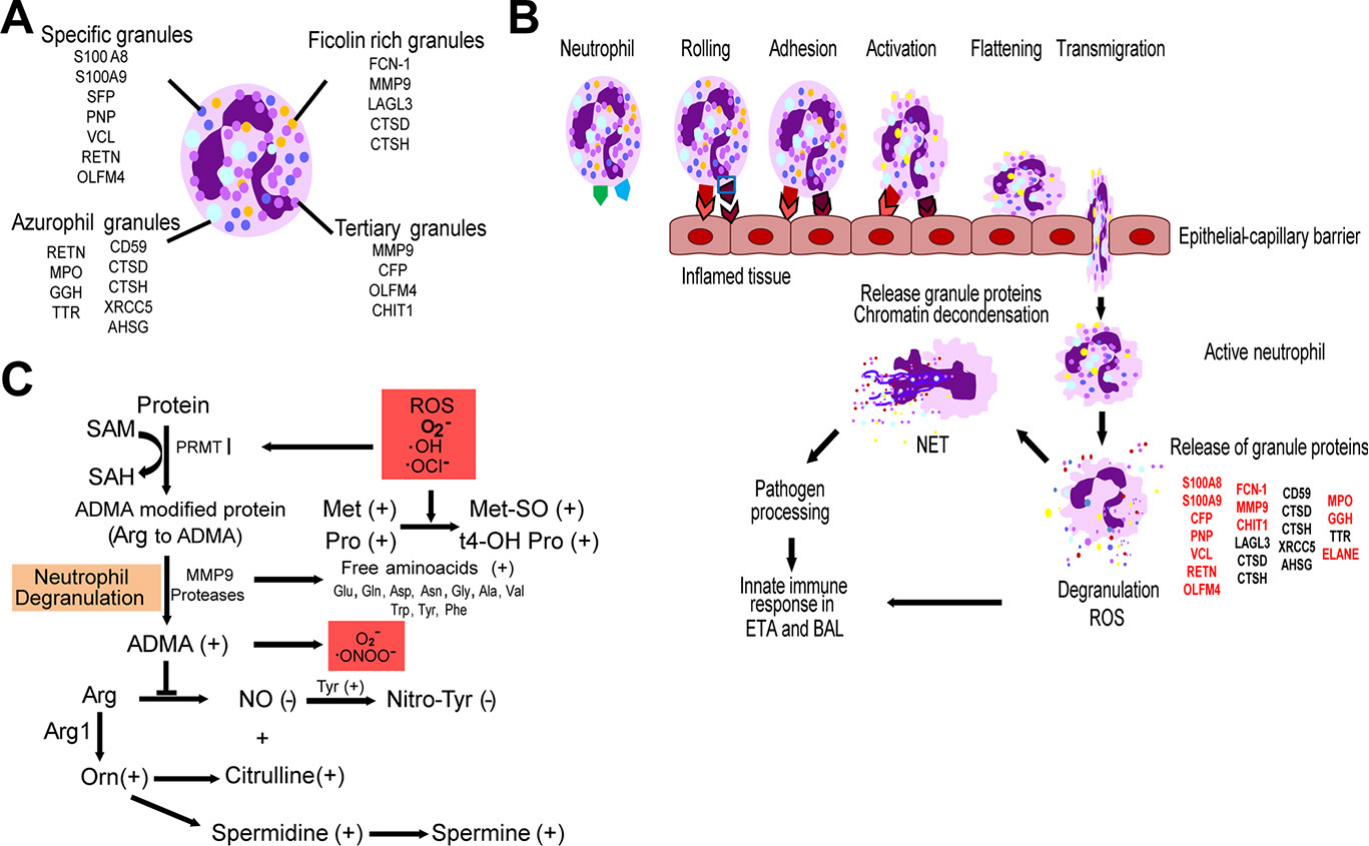
**Proposed mechanisms of neutrophil-mediated innate immune responses in the respiratory tract upon VAP infection.***A*, Neutrophil granules and their proteins detected in ETA and BAL. *B*, Recruitment of neutrophils at the site of inflamed trachea and alveoli in response to pathogen stimuli. Neutrophil rolling, adhesion and transmigration into inflamed alveoli and tracheal tissues are facilitated by VCL, ACTN1, MYYH9, and MYH12. Activation of neutrophils can induce degranulation and release granule proteases, oxidases, peroxidases and other antimicrobial proteins for pathogen processing (53). *C*, Proposed metabolic fate of neutrophil degranulation and reactive oxygen species (ROS)-induced oxidative stress in the respiratory tract during VAP infection.

Similar to the proteomic analysis, Bland-Altman analysis showed no significant bias of metabolites in ETA and BAL matrices as the majority of data were within the limits of agreement for 95% confidence interval (mean difference of 0, upper limit of 4.7 and lower limit of −4.8) (Fig. 5*A*). The unsupervised temporal clustering using median metabolite concentrations showed two distinct clusters of *Baseline* and post-*Baseline* samples. Unlike the temporal clustering of the ETA proteome, *Control* clustered more closely with *Post-VAP*. This suggests that at the time of intubation, ETA metabolomes of both control and VAP patients were similar and changed as infection progressed from *pre-VAP, VAP positive* to *post-VAP*. The clustering of *Control* with *post-VAP* may highlight an effect of residual inflammation because of intubation instead of infection (Fig. 5*B*). We saw increased concentration of several metabolites at either *pre-VAP* or *VAP positive,* or in both. These metabolites were decreased in both *Control* and *Post-VAP*. These changes may reflect patient responses against infection. Further comparison of *VAP positive* and *Baseline* ETA identified an increase in 53 metabolites and a decrease in one glycerophospholipid (PC aa C30:0) upon VAP infection (*p* < 0.05, fold change >±2) (Table VI). The high concentrations of amino acids and t4-OH Pro in *VAP positive* ETA may have resulted from the activity of neutrophil proteases, matrix metalloproteinase-9 (MMP9) and ELANE, whereas increased concentration of Met (*p* < 0.05) and its oxidative product (Met-SO, *p* = 0.977) may indicate reactive oxygen species (ROS) formation by MPO, NADH oxidase or PNP during neutrophil degranulation (Fig. 7*C*). In *VAP positive* ETA, we also observed a 2- to 5-fold increase of five polyamines as products of arginine catabolism, *i.e.* ADMA, ornithine, spermine, and spermidine (*p* < 0.05) and their precursor polyamine (citrulline, *p* = 0.067). Elevated ADMA points toward ROS induced proteolysis of methylated proteins (Fig. 7*C*). Its inhibitory action on nitric oxide synthase (NOS) was measured as a decrease in the ratio of Nitro-Tyr (*p* = 0.371) to Tyr (*p* < 0.05), (Fig. 7*C*) also previously reported in community-acquired pneumonia (32). We detected significantly increased acylcarnitines, glycerophospholipids and sphingolipids during VAP infection. Similar trends of plasma lipid metabolism have been reported in VAP patients (33).

**Table VI:** Differentially abundant metabolites between Baseline and the day of clinical diagnosis (VAP positive)

Type	Metabolite	log2(FC)^a^	*p* Value
Acylcarnitines	C2	1.42	0.032
Acylcarnitines	C3	1.10	0.032
Acylcarnitines	C4	1.61	0.001
Acylcarnitines	C6 (C4:1-DC)	1.02	0.024
Amino acids	Ala	2.17	0.007
Amino acids	Gln	1.39	0.014
Amino acids	Glu	2.22	0.032
Amino acids	Gly	2.16	0.005
Amino acids	His	2.24	0.007
Amino acids	Met	2.79	0.010
Amino acids	Orn	2.72	0.019
Amino acids	Trp	1.97	0.007
Amino acids	Tyr	2.92	0.005
Amino acids	Val	2.90	0.007
biogenic amines	ADMA	2.12	0.014
biogenic amines	Spermidine	1.07	0.014
biogenic amines	Spermine	2.32	0.010
biogenic amines	t4-OH-Pro	1.17	0.009
biogenic amines	Met-SO	1.49	0.977
biogenic amines	Citrulline	2.19	0.067
biogenic amines	Ornithine	2.72	0.018
Glycerophospholipids	PC aa C30:0	−1.23	0.019
Glycerophospholipids	PC aa C32:3	1.25	0.025
Glycerophospholipids	PC aa C36:1	1.08	0.042
Glycerophospholipids	PC aa C36:2	1.74	0.032
Glycerophospholipids	PC aa C36:3	1.76	0.042
Glycerophospholipids	PC aa C38:0	1.15	0.024
Glycerophospholipids	PC aa C38:3	2.19	0.024
Glycerophospholipids	PC aa C38:4	2.33	0.024
Glycerophospholipids	PC aa C38:5	1.58	0.032
Glycerophospholipids	PC aa C40:2	1.07	0.014
Glycerophospholipids	PC aa C40:3	1.25	0.037
Glycerophospholipids	PC aa C40:5	1.54	0.032
Glycerophospholipids	PC aa C40:6	1.26	0.014
Glycerophospholipids	PC aa C42:1	1.43	0.005
Glycerophospholipids	PC ae C34:1	1.23	0.007
Glycerophospholipids	PC ae C34:2	1.59	0.014
Glycerophospholipids	PC ae C34:3	1.23	0.032
Glycerophospholipids	PC ae C36:1	1.19	0.019
Glycerophospholipids	PC ae C36:2	1.37	0.014
Glycerophospholipids	PC ae C36:3	1.86	0.005
Glycerophospholipids	PC ae C36:4	1.49	0.019
Glycerophospholipids	PC ae C38:1	1.78	0.032
Glycerophospholipids	PC ae C38:2	1.98	0.010
Glycerophospholipids	PC ae C38:3	1.88	0.019
Glycerophospholipids	PC ae C38:4	2.02	0.019
Glycerophospholipids	PC ae C38:5	1.78	0.032
Glycerophospholipids	PC ae C40:1	1.73	0.014
Glycerophospholipids	PC ae C40:2	1.33	0.042
Glycerophospholipids	PC ae C40:3	1.70	0.016
Glycerophospholipids	PC ae C40:4	1.12	0.008
Glycerophospholipids	PC ae C40:5	2.39	0.019
Glycerophospholipids	PC ae C40:6	2.32	0.010
Glycerophospholipids	PC ae C42:2	2.41	0.033
Glycerophospholipids	PC ae C42:4	3.46	0.013
Glycerophospholipids	PC ae C44:6	1.20	0.042
Sugars	H1	1.69	0.003


a Log2(FC) of *VAP* positive *versus Baseline* in VAP patients; *p-value* determined using Wilcoxon rank sum test, *p* < 0.05 is considered as significant.

##### VAP Pathogen Specific Peptide Signatures Are Found in ETA

We identified a total of 66 unique microbial peptides (supplemental Table S6) corresponding to 59 proteins specific to VAP pathogens (Listed in supplemental Table S1) (in ETA and BAL of 15 patients (5 control and 10 cases). Of these, 24 peptides were associated to Gram-positive, 38 to Gram-negative bacteria, and 4 to Gram-positive yeast. In ETA, 62 peptides out of 66 were detected in VAP patients compared with 8 peptides (shared with VAP) in control group. Only 10 peptides were identified in BAL, 3 of which were uniquely observed in BAL (supplemental Table S6). Although low incidence and abundance of microbial peptides in both ETA and BAL precluded any quantitation, we evaluated their association with VAP status. Peptides associated with Gram-positive and negative bacteria were found in ETA from both VAP and control groups, however, peptides counts were substantially elevated in VAP (144 peptides) in contrast to controls (14 peptides). Gram-negative pathogens were more predominantly observed in VAP patients across most of the intubation days, as indicated by their peptide distributions (Gram-negative, 82; Gram-positive, 37; yeast, 25 peptide counts). These peptides were detected at least 1 day prior to VAP diagnosis in most of the patients (supplemental Fig. S2).

**Fig. 6:**
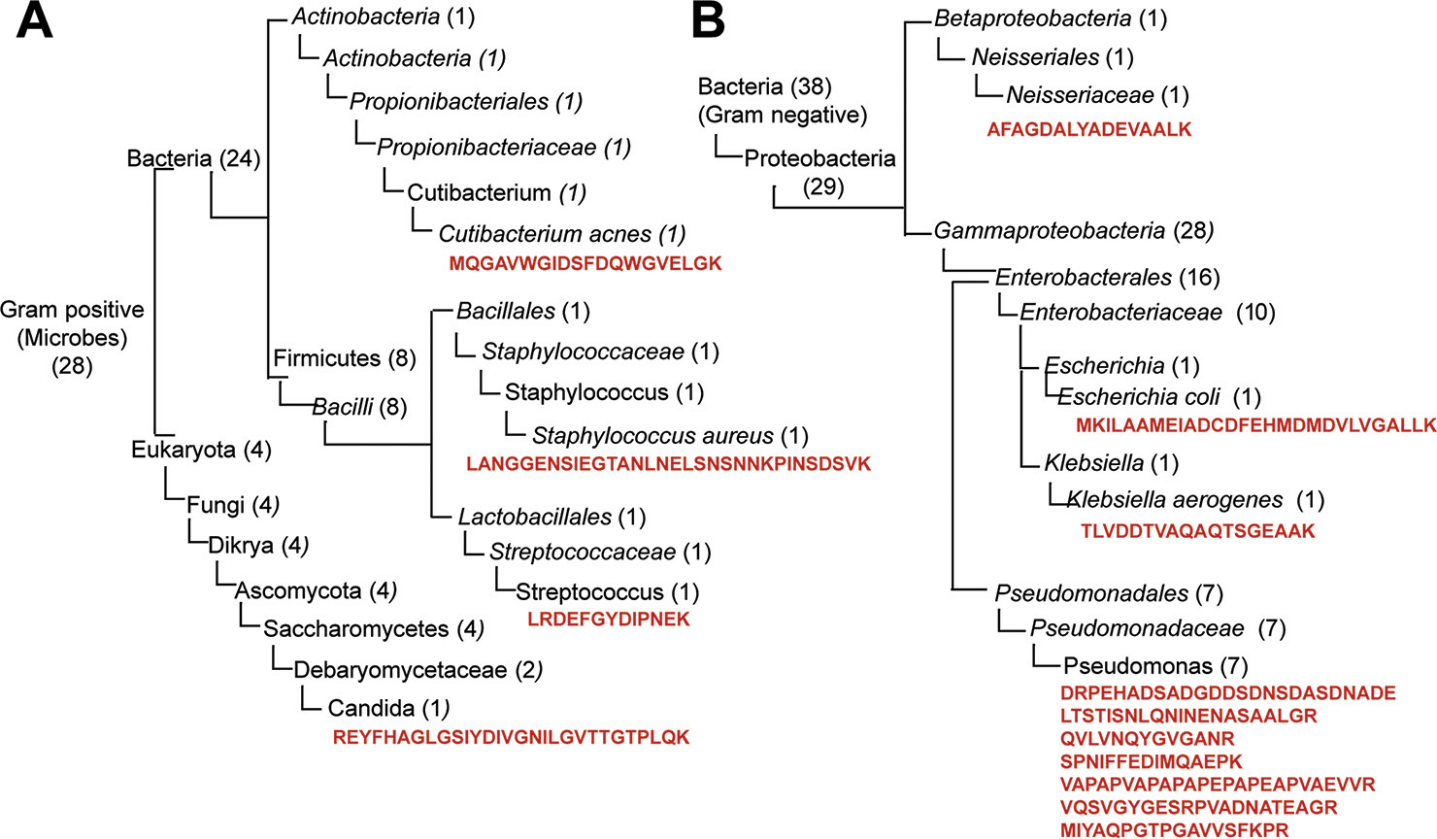
**Taxonomic characterization of VAP pathogens using metaproteome approach.** Dendogram of Gram-positive VAP pathogen (*A*) and Gram-negative VAP pathogens (*B*). In parenthesis the number of peptides identified at each level. The peptide sequence specific to lowest common ancestor are represented in red.

Using UniPept, we confirmed the specificity of the microbial peptides at family, genera and/or species level and performed peptide-based taxonomic classification (Fig. 6). Twelve peptides belong to Gram-positive bacteria, 8 of which represent *Bacilli* and 1 is associated with actinobacteria (supplemental Table S7, Table VII). Three were classified to Staphylococcus, Streptococcus and Cutinibacterium genera and 2 peptides exhibit species level specificity, 1 for *Staphylococcus aureus* and 1 for *Cutinibacterium acnes* (Fig. 6*A*, supplemental Table S7). Four peptides are associated with 4 yeast proteins, one of which is specific to Candida. We also identified 29 peptides linked to Gram-negative proteobacteria (supplemental Table S7). Most of these peptides are associated with *Enterobacteriaceae, Pseudomonadaceae* and *Neisseriaceae* families of VAP pathogens, 7 of which are associated to Pseudomonas, 1 to *Escherichia coli* and 1 to *Klebsiella aerogenes* (Fig. 6*B*, Table VII).

**Table VII:** Species-specific peptides leading to identification of bacterial proteins in ETA

Peptides Sequence	Uniprot Accession	Gene ID	Specificity to VAP Pathogens	Gram Nature
LANGGENSIEGTANLNELSNSNNKPINSDSVK	A0A0H2 × JB4	Ear	*Staphylococcus aureus*	Positive
LRDEFGYDIPNEK	A0A0T8BKI5	gmuF	Streptococcus	Positive
MQGAVWGIDSFDQWGVELGK	W4TV09	JCM18916	*Cutibacterium acnes*	Positive
REYFHAGLGSIYDIVGNILGVTTGTPLQK	B9WKF6	CD36_72570	Candida	Positive
MKILAAMEIADCDFEHMDMDVLVGALLK	A0A1M0KRG1	BK251_25065	*Escherichia coli*	Negative
TLVDDTVAQAQTSGEAAK	A0A094ZQ80	ASV18	*Klebsiella aerogenes*	Negative
DRPEHADSADGDDSDNSDASDNADE	Q4KJ62	rpsF	Pseudomonas	Negative
LTSTISNLQNINENASAALGR	A0A0K1QPN3	B723_13770	Pseudomonas	Negative
QVLVNQYGVGANR	A0A1U9LQW5	oprF	Pseudomonas	Negative
VAPAPVAPAPAPEPAPEAPVAEVVR	A0A1U9LQW5	oprF	Pseudomonas	Negative
VQSVGYGESRPVADNATEAGR	A0A1U9LQW5	oprF	Pseudomonas	Negative
MIYAQPGTPGAVVSFKPR	A0A0T8KUT8	acoD	Pseudomonas	Negative
SPNIFFEDIMQAEPK	A0A1U9LRJ1	pedI	Pseudomonas	Negative

## DISCUSSION

The present study describes VAP mediated host re-sponses in ETA and BAL of 16 intubated patients. This is also the first detailed characterization of the ETA proteome and metabolome. We detected 3067 unique proteins in ETA sampled longitudinally, compared with 1139 proteins in BAL. We also observed a >10% increase in unique BAL proteins compared with previous studies (16, 34). Despite our observation of a 3-fold higher proteome diversity and although less invasive, ETA has been historically overlooked in favor of BAL (35). Our study revealed that ETA is functionally diverse and highly enriched in proteins involved in innate and adaptive immunity, suggesting that it is an attractive source to study lung infection. In VAP patients, we observed upregulation of inflammation, ROS formation and neutrophil-mediated innate immunity in the respiratory tract during VAP infection, leading to pathogen processing (Fig. 7). Fig. 7*A* represents neutrophil granule proteins detected in ETA. The elevated levels of vinculin and myosins may imply extracellular matrix (ECM) adhesion and migration of neutrophils (Fig. 7*B*) (36, 37). Increased abundance of pathogen recognition molecules ficolin-1 and properdin in ETA may be linked to complement system activation via interaction with bacterial polysaccharides (38). In support of this, antimicrobial neutrophil proteins S100A8 and S100A9, with proinflammatory and chemotactic activity (39), were highly elevated during VAP infection. Furthermore, the VAP-associated increase in granule proteins, such as chitotriosidase-1, gamma-glutamyl hydrolase, resistin, olfactomedin-4, and MMP9 (40, 41) all point toward inflammation and neutrophil degranulation to promote pathogen clearance (Fig. 7*B*). Li and colleagues predicated an increase in plasma MMP9 with the severity and progression of VAP infection (42). During infection, release of these hydrolytic granule proteins upon neutrophil degranulation may have detrimental effect on ECM of airway epithelium. The high concentrations of free amino acids as well t4-OH Pro in ETA (Fig. 7*C*) suggest altered ECM integrity through collagen degradation. Effect of neutrophil metalloproteases on ECM modulation has been previously studied (43). The neutrophils are key players of pathological inflammation in lung infections such as VAP (44). In our study, we observed increased lung inflammation and neutrophil degranulation during VAP infections, which may promote formation and release of neutrophil extracellular traps (NETs). The role of NETs in VAP pathogenesis has been recently investigated in BAL of 100 critically ill patients (45).

Although, NETosis provides a defense network against pathogen infection, studies have shown that exaggerated NETs can be detrimental to the lung environment (46). Considering the tissue protective effect of polyamines (47), their high levels at VAP infection may have role in tissue protection as a balancing mechanism against adverse impact of neutrophil degranulation on lung epithelium.

Overall, ETA investigation suggests a cascade of neutrophil degranulation events starting with neutrophil recruitment, adhesion, and migration toward the site of infection. Induction of oxidative stress activates degranulation and chromatin decondensation as a host response against VAP. Validation of elevated ELANE and MPO by ELISA confirmed the role of neutrophil degranulation in early host response to VAP. Studies have demonstrated higher specificity (93 to 96%) and sensitivity (75 to 91%) of serum CRP at levels ranging from 48 to 200 mg/L in diagnosing pneumonia (48, 49). The presence and role of CRP and NME1-2 only at *VAP positive* is still unclear and warrants further investigation. We also looked for other inflammatory markers such as interleukin-1β, interleukin-8, soluble triggering receptor expressed on myeloid cells type 1, procalcitonin, and the mid-region fragment of pro-adrenomedullin in ETA from VAP and control patients. We detected interleukins in ETA of both VAP and control patients (supplemental Table S9). However, none of these makers were significantly different between *Baseline* and *VAP*.

We observed the modulation of protein catabolism, ROS synthesis, polyamine and lipid metabolism in VAP ETA (Fig. 7*C*). The formation of ROS was measured using protein inducers (MPO and purine nucleoside phosphorylase) and was confirmed by metabolic indicators (Met-SO, t4-OH-Pro and ADMA) (30, 50, 51). Metzler *et al.* have further shown ROS-triggered translocation of ELANE to the nucleus and subsequent induction of chromatin decondensation. Oxidative stress is characteristic of neutrophil degranulation and has been reported in other pulmonary diseases (52). Our hypothesis is corroborated through the investigation of longitudinal ETA sampling, which has identified host innate immune mechanism of pathogen processing and provided enhanced granularity of VAP progression.

In our study, we employed a quantitative and qualitative BAL culture, a clinical standard for VAP diagnosis to identify VAP pathogens. Our metaproteomic strategy identified candidate peptides exhibiting specificity at family, genus and species levels for VAP pathogens. Along with clinical symptoms, pathogen detection has a crucial role in VAP diagnosis and antibiotic treatment alignment. Most patients were on broad spectrum antibiotics throughout (standard of care) and beyond the end of the ventilation period. Our study was mainly focused on dissecting VAP using ETA and BAL, the collection of any samples post ventilation was not feasible to determine antibiotic treatment outcome. Although, we did not correlate successful antibiotic treatment with disappearance of pathogen peptides, we did investigate the association of peptides in relation to VAP infection. The Gram-negative bacterial peptides were detected in most patients' ETA over multiple days with at least 1 day prior to VAP diagnosis.

Because microbial peptide abundance was low compared with host proteome, quantitation was beyond the scope of this study. Future studies will need to evaluate the proteotypic and quantotypic properties of these microbial peptides and their use in VAP diagnosis.

Intubation is one of the most common interventions in critical care and has been linked to increased susceptibility of lung infection and mortality. Intubation procedure, length of stay and inappropriate antibiotic treatment, as well as preexisting conditions such as compromised or weakened immunity may contribute to microbial dysbiosis and development of pneumonia. Ultimately, these alterations to the host environment may be reflected at the pulmonary interface. Our study has focused on ETA to conduct a molecular survey of upper airways during VAP development and progression. We have shown that ETA is reflective of a rich and diverse airway proteome. In VAP, we identified an early upregulation of immune-modulatory proteins associated with an early host response to infection. We also looked for VAP pathogen peptides in ETA, and detected unique, species-specific peptides correlated with cultures. In the majority of VAP patients, these distinctive pathogen signatures were present at least 1 to 2 days earlier than the BAL culture-based diagnosis. Although presenting distinctive features from BAL, ETA may be an attractive alternative for earlier and cost-effective clinical diagnosis of pneumonia in intubated patients.

## DATA AVAILABILITY

The MS proteomics data have been submitted to ProteomeXchange Consortium via the PRIDE partner repository with data set identifier PXD010715. The normalized data for metaproteomics (supplemental Table S6–S7), quantitative metabolomics (supplemental Table S8) and host proteomics (supplemental Table S9) are also available in supplemental excel files.
